# Recent progresses on single-atom catalysts for the removal of air pollutants

**DOI:** 10.3389/fchem.2022.1039874

**Published:** 2022-10-28

**Authors:** Yang Wang, Min Wang

**Affiliations:** ^1^ State Key Laboratory of High Performance Ceramics and Superfine Microstructure, Shanghai Institute of Ceramics, Chinese Academy of Sciences, Shanghai, China; ^2^ Center of Materials Science and Optoelectronics Engineering, University of Chinese Academy of Sciences, Beijing, China

**Keywords:** single-atom catalysts, air pollutant removal, catalytic oxidation, carbon monoxide, volatile organic compounds, nitrogen oxides

## Abstract

The booming industrialization has aggravated emission of air pollutants, inflicting serious harm on environment and human health. Supported noble-metals are one of the most popular catalysts for the oxidation removal of air pollutants. Unfortunately, the high price and large consumption restrict their development and practical application. Single-atom catalysts (SACs) emerge and offer an optimizing approach to address this issue. Due to maximal atom utilization, tunable coordination and electron environment and strong metal-support interaction, SACs have shown remarkable catalytic performance on many reactions. Over the last decade, great potential of SACs has been witnessed in the elimination of air pollutants. In this review, we first briefly summarize the synthesis methods and modulation strategies together with the characterization techniques of SACs. Next, we highlight the application of SACs in the abatement of air pollutants including CO, volatile organic compounds (VOCs) and NO_x_, unveiling the related catalytic mechanism of SACs. Finally, we propose the remaining challenges and future perspectives of SACs in fundamental research and practical application in the field of air pollutant removal.

## 1 Introduction

Air pollutants, such as carbon monoxide (CO), volatile organic compounds (VOCs), nitrogen oxides (NO_x_) and so on, are very hazardous to environment and human health (Liu Z. S. et al., 2019; He et al., 2019; Chen et al., 2021; Dong et al., 2021; He et al., 2021; Peng et al., 2021). With the surging industrialization, ever-growing emission of air pollutants has attracted great attention. Catalytic oxidation can transform diverse air pollutants into non-toxic or less harmful substances at room or low temperature, which is deemed promising to overcome gaseous pollutant problem. Catalytic oxidation techniques can be categorized into thermo-catalysis, photocatalysis and photothermal catalysis. Thermo-catalysis requires high temperature to activate oxygen species to oxidize air pollutants; and photocatalysis can produce reactive radicals by photo-generated carriers, which are responsible for degradation of air pollutants. Photothermal catalysis is a novel technique coupling with the advantages of thermo-catalysis and photocatalysis, exhibiting higher activity than individual process. Supported noble-metal catalysts, such as Au, Pt, Pd, etc., are the most widely used materials for air pollutant elimination owing to their high catalytic performance (Bai et al., 2019; Zhang Y. Y. et al., 2019; Gan et al., 2019; Lu et al., 2019; Chen H. X. et al., 2020; Yang et al., 2021; Li J. T. et al., 2022; Chen et al., 2022). Traditionally, most noble-metals are supported as nanoparticles or clusters, but only a small portion of metal atoms can work as active sites, giving low atom utilization (Shang et al., 2021). Besides, high cost is also an obstacle to their large-scale applications. Therefore, tremendous efforts have made to further improve the atom utilization and reduce the consumption of noble-metals.

In 2011, Zhang et al. first defined the concept of single-atom catalysts (SACs) for isolated single Pt atoms anchored to the surface of FeO_x_ (Pt_1_/FeO_x_), whose TOF for CO oxidation was ∼2–3 times higher than that on Pt nanoparticles loaded on FeO_x_ (Qiao et al., 2011). Henceforth, single-atom catalysis becomes an up-growing topic and has grown into a research hotspot in heterogeneous catalysis. The full exposure of active sites maximizes the atom utilization, accomplishing atomic economy and resource conservation of noble metals (Zhang L. et al., 2019; Zhou et al., 2020; Li Z. et al., 2022). As distinct from nanoparticles, unique coordination environments and electronic structures of SACs endow them excellent activity and selectivity (Jiang and Wang, 2018; Gu Y. et al., 2021; Zhang et al., 2021).

The catalytic performance of supported metal catalysts greatly relies on the properties of supports. In 1978, Tauster et al. developed the concept of strong metal-support interaction (SMSI) to depict the phenomenon that Pt nanoparticles were encapsulated by sub-oxide TiO_2-x_ species deriving from TiO_2_ support (Tauster et al., 1978). In addition to the migration and reconstruction of chemical components, metal-support interactions (MSI) are frequently accompanied by electron transfer and interfacial effects. Hence, MSI are expanded into strong metal-support interaction (SMSI), electronic metal-support interaction (EMSI) and interfacial perimeter in supported metal-nanoparticle catalysts. Due to the ununiform distribution of metal nanoparticles and complex interfaces, it is very challenging to quantitatively clarify the nature of the MSI effects. While, in SACs, the well-defined single-atom site provides a good model to remove these barriers. When the supported metal exists as single atom, there are no complex interfaces between metals and supports. The catalyst systems have been largely simplified. The MSI originating from the chemical bonding between metal single-atoms and supports can be maximized to significantly influence their catalytic performances. The coordination bonding between metal atoms and supports can stabilize the isolated metal atoms with high surface energy to prevent their migration and aggregation. The EMSI, resulting from the difference in chemical potentials of metals and supports, will induce the charge redistribution of the whole SAC systems, thus modulating the adsorption and activation behaviors towards reactants and intermediates and enhancing their catalytic performances (Li et al., 2019; Wan X. et al., 2022).

Thanks to their unequalled advantages, SACs have shown conspicuously improved performance on a range of reactions in energy and environment fields (Chen Y. J. et al., 2018; Weon et al., 2020). For the abatement of air pollutants, the application of SACs has extended from initial CO to other pollutants, e.g., formaldehyde (HCHO), benzene (C_6_H_6_), toluene (C_7_H_8_), NO_x_ and so on. For instance, the single Ag atoms anchored at the tunnel openings of the hollandite-type manganese oxide (HMO) were fabricated by Huang and co-workers (Huang et al., 2012), on which the TOF of HCHO oxidation was 7 times higher than that on Ag nanoparticles loaded on HMO. The high activity of Ag single-atoms was ascribed to the enhanced activation ability of both lattice oxygen and molecule oxygen. Subsequently, Ag_1_/HMO was also demonstrated to be reactive for benzene oxidation (Chen et al., 2017c). Hence, SACs have been demonstrated to be appealing for the catalytic removal of air pollutants. In the last decade, SACs have achieved great progresses on the catalytic oxidation of air pollutants and it is very necessary and highly desired to make a systematic summary towards current advances.

**FIGURE 1:**
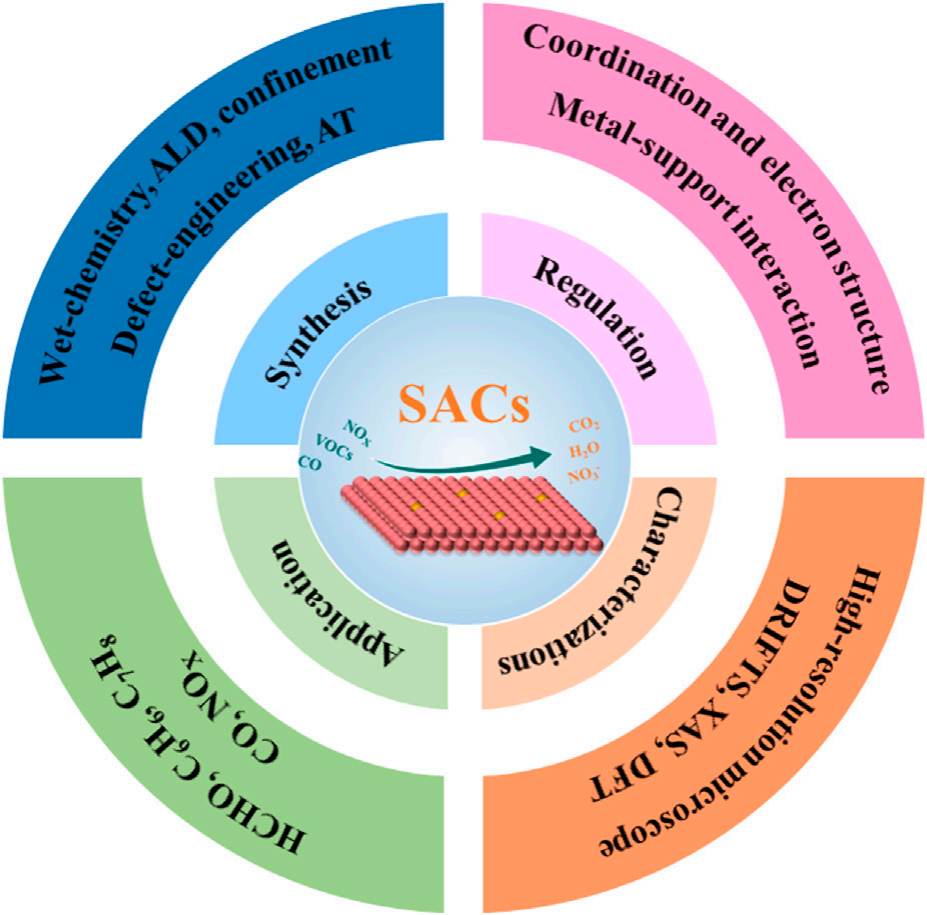
Schematic illustration of single-atom catalysts for air pollutant removal.

In this review, we first illustrate the synthesis and modulation methods together with the characterization techniques of SACs. Further, we focus on the application of SACs in elimination of air pollutants and summarize the corresponding intrinsic catalysis mechanism. More importantly, the structure-function relationship of SACs is discussed to give a hand for the rational design of SACs. Finally, the challenges and prospects of future research on SACs for elimination of air pollutants are provided (Figure 1).

## 2 Synthesis of single-atom catalysts for air pollutant removal

The isolated atoms tend to agglomerate to form nanoparticles or nanoclusters due to its high surface energy. So, how to prevent the agglomeration of single atoms is the main challenge in the fabrication of SACs. Recently, variety of methods have been developed to synthesize SACs, such as wet-chemistry precipitation, atomic layer deposition (ALD), spatial confinement, defect-engineering and atom trapping (AT), etc.

The wet-chemistry method, including coprecipitation and impregnation, is easy to be operated without special equipment (Pei et al., 2017; Cao et al., 2020; Sun et al., 2020; Muravev et al., 2021; Hai et al., 2022). The crucial point is to control the metal content and select appropriate support. Pt_1_/FeO_x_ was successfully built by a co-precipitation method (Qiao et al., 2011); FeO_x_ support with large surface area were used and low amount (0.17 wt%) of Pt was loaded, ensuring atomic dispersion of Pt single atoms. However, increased loading amount of metal atoms is a big issue for wet-chemistry method. Notably, Hai and co-workers (Hai et al., 2022) introduced a two-step annealing approach to realize ultra-high-density (UHD) of SACs with metal content up to 23 wt%. Regulating the bonding of metal precursors to supports by stepwise ligand removal can prevent their thermally induced aggregation into nanoparticles. The annealing temperature (T_1_) of the first step should be lower than the decomposition temperature of metal precursors and the annealing temperature (T_2_) of the second step should be high enough to remove the remaining ligands and transform the chemisorbed metal precursors into UHD-SACs.

The atomic layer deposition (ALD) is a promising tool to achieve large-scale synthesis of SACs. The ALD process to fabricate SACs includes two main steps: metal precursors react with adsorbed oxygen on support surface; then, metal precursor ligands are oxidized to M-O species (M: metal atoms) by oxygen pulse, resulting in the formation of single atoms (Zhang L. et al., 2018). Sun’s group (Sun et al., 2013) first synthesized isolated Pt atoms anchored to graphene nanosheets using MeCpPtMe_3_ as the precursor and O_2_ as counter reactant by ALD techniques. Pt single-atoms loaded on Co_3_O_4_, CeO_2_ and ZrO_2_ were also prepared using ALD methods (Li et al., 2019). The metal oxides were exposed in MeCpPtMe_3_ precursor and O_3_ was adopted to burn off the ligand of MeCpPtMe_3_ to obtain Pt SACs. The content of Pt can be tuned *via* changing exposure time of MeCpPtMe_3_ and also ALD cycle numbers. However, metal atoms are apt to be adsorbed on the deposited metal atoms to aggregate into cluster, thus, high metal loading is difficult to be achieved.

Defects on support, such as cation vacancies, oxygen vacancies and step edges etc., are good candidate sites to anchor metal precursors; then enhancing charge-transfer interaction between single atoms and defective sites can stabilize isolated metal atoms to restrict their migration. Chen and co-workers (Chen J. et al., 2018) created Mn vacancies by the redox reaction between MnO_2_ and H_2_O_2_ in acidic medium, and redox reaction as follows: MnO_2_ (s) + H_2_O_2_ (aq) + 2H^+^ (aq) = Mn^2+^ (aq) + 2H_2_O (l) + O_2_ (g). Later, Au atoms were anchored on Mn vacancies. This strategy can be extended to prepare SACs using other oxides as supports (Xia et al., 2018; Xia et al., 2019; Yi et al., 2020). Ni^2+^ vacancies in Ni(OH)_x_ also had strong stabilizing effect on single-atomic Pt species (Zhang J. et al., 2018). Besides, Li’s group (Wan et al., 2018) reported oxygen vacancies of TiO_2_ stabilizing Au atoms through Ti-Au-Ti structure. Except for the above common defects, the step edges of CeO_2_ can also anchor Pt single-atoms to form PtO_4_ units (Dvorak et al., 2016).

The spatial confinement of pore structure has been found to be capable of achieving spatial segregation of metal precursors; then, the single atoms are generated after ligand removal. Zeolite is considered as good host materials to encapsulate isolated metal atoms due to its abundant microporous channels (Shan et al., 2017; Liu Y. W. et al., 2019; Liu et al., 2020). Tang et al. (Tang et al., 2018) introduced Rh cations to the internal surface of micropores of ZSM-5 through a method integrating vacuum pumping and incipient wetness impregnation. EXAFS results disclosed that Rh single atoms were dispersed in micropores of ZSM-5 by Rh_1_O_5_ coordination structure. Metal-organic frameworks (MOFs) with regular pore structures also have great potential to separate metal precursor (Chen Y. J. et al., 2017; Wang et al., 2018). For example, the cage of ZIF-8 can package Ru (acac)_3_ precursor, because the molecular size of Ru (acac)_3_ (9.7 Å) is smaller than the cavity diameter (11.6 Å) and larger than the pore diameter (3.4 Å) of ZIF-8 (Ji et al., 2019). Ru single-atoms were produced after removing ligand of Fe (acac)_3_ by thermal reduction treatment below the thermal decomposition temperature of ZIF-8.

**FIGURE 2:**
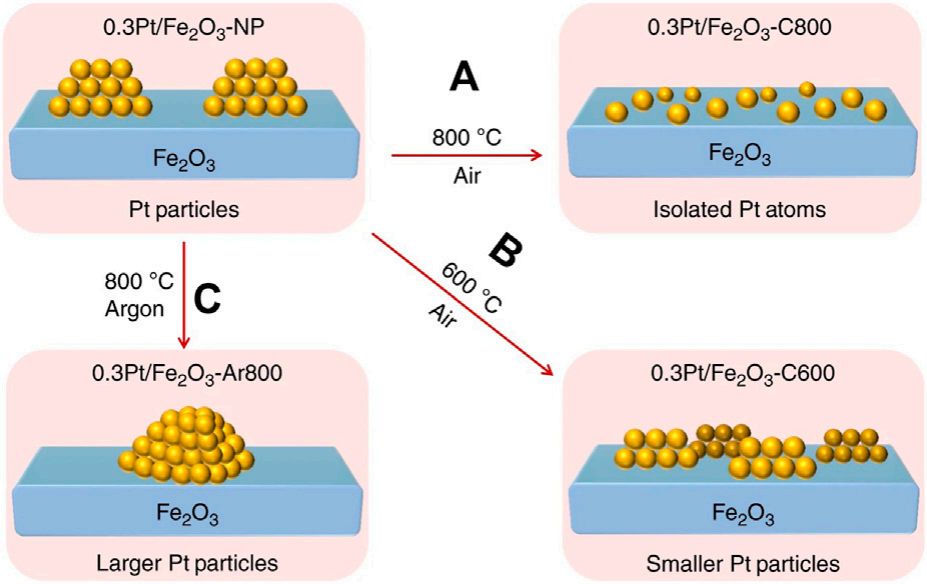
Atom trapping method at high temperature to synthesize Pt/Fe_2_O_3_ SACs (NPs: nanoparticles; C800: calcining at 800°C in air; Ar800: calcining at 800°C in Ar; C600: calcining at 600°C in air). Reprinted from Lang et al. (2019). Copyright ^©^ 2019, with permission from Springer Nature.

Atom trapping (AT) can directly transform metal nanoparticles to single atoms (Xiong et al., 2017; Jiang et al., 2020; Pham HN et al., 2022; Zhuang et al., 2022). Datye et al. (Jones et al., 2016) found that Al_2_O_3_ supported Pt nanoparticles converted into Pt single-atoms on CeO_2_ when the physical mixtures of Pt/Al_2_O_3_ and CeO_2_ were aged in air at 800°C. This is due to that volatile PtO_2_ was emitted from Pt nanoparticles and trapped by CeO_2_ support during the heat process. Meanwhile, Fe_2_O_3_ substrate is also able to capture volatile PtO_2_ units to generate Pt/Fe_2_O_3_ SACs in air at 800°C (Figure 2A) (Lang et al., 2019). But the smaller nanoparticles were formed at lower temperature (Figure 2B) and the larger nanoparticles were emerged in Ar (Figure 2C). Thus, AT methods need three basic conditions: a high temperature to break metal-metal bond to produce mobile Pt species; an oxidizing atmosphere to produce volatile PtO_2_ units; a strong interaction between support and Pt species to anchor Pt single atoms.

## 3 Regulation of single-atom catalysts for air pollutant removal

The nature of SACs depends on their unique coordination environment and electron structure, as well as their interactions with the support (Jiang et al., 2021a), so the regulation of SACs mainly focuses on these aspects. Unfortunately, in the field of air pollutant purification, regulations on SACs have rarely been investigated, so we just briefly discuss the current status in this section.

The selection and modification of supports has great impact on the catalytic behavior of SACs. The reducibility of supports can alter the charge state of Au single-atoms and promote its stability (Liu et al., 2017). On irreducible supports, e.g., MgO, Al_2_O_3_ and ZrO_2_ etc., Au single atoms exhibit little electron transfer and weak bonding to CO. In contrast, on reducible supports, e.g., CeO_2_, TiO_2_ and Fe_2_O_3_, charge transfer from Au single-atoms to supports generates positively charged Au^+^ species, which are very stable upon CO adsorption. Heteroatom-doping can adjust the property of supports to improve the activity of SACs (Zhou X. et al., 2022; Meng et al., 2022). The CO oxidation activity of Pd/CeO_2_ can be enhanced after Pr doping (Deng et al., 2022), which reduces the formation energy of oxygen vacancies around Pd sites, promotes the dissociation of O_2_ and reaction rate of CO oxidation. Hydrogen reduction treatment is an effective path to tune the properties of supports. Gao et al. (Gao et al., 2022) found that reduced TiO_2_ was conducive to the generation of Pt single atoms with high electron deficiency, which can suppress the strong CO adsorption and profit the adsorption/activation of O_2_.

**FIGURE 3:**
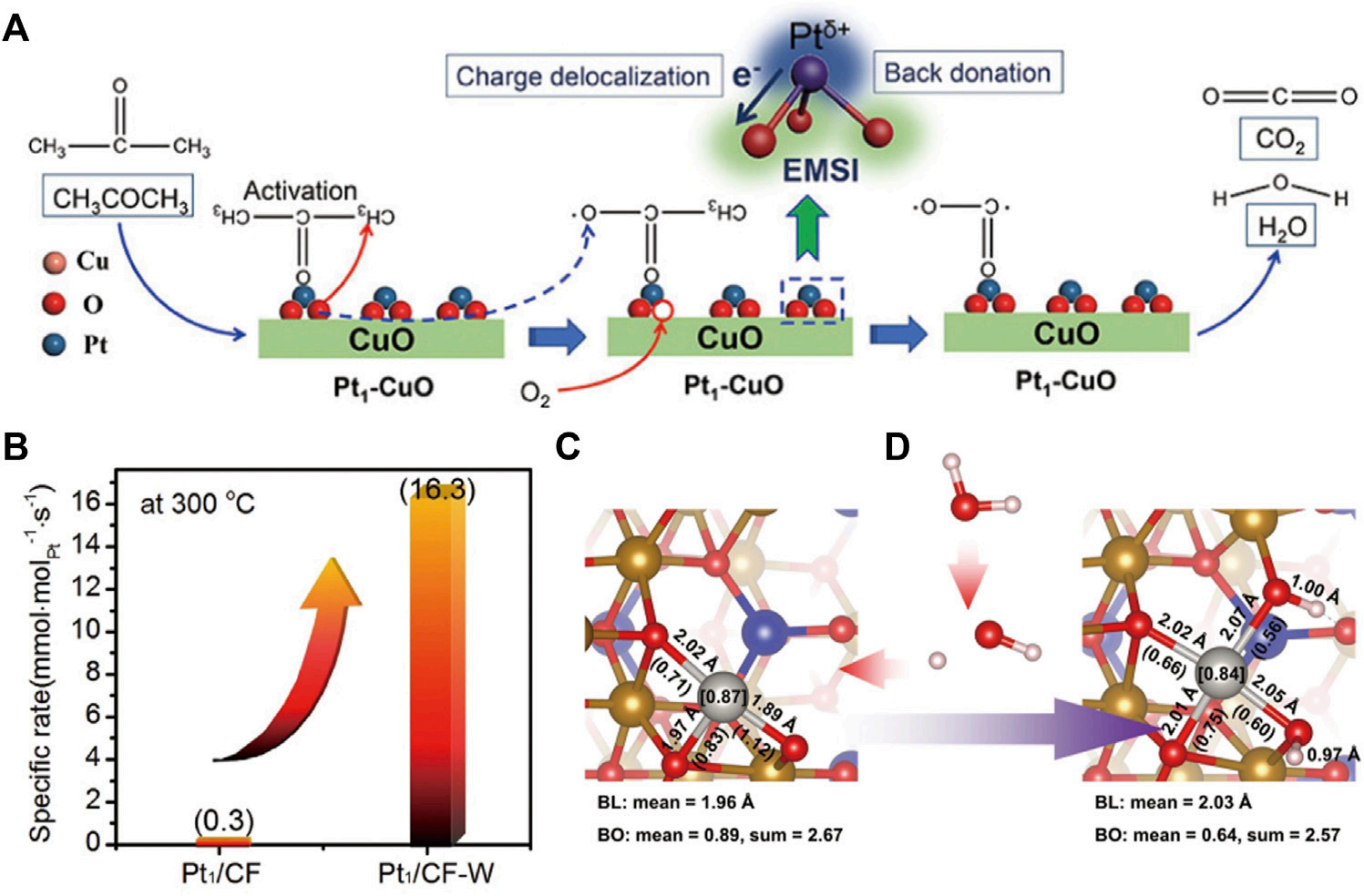
**(A)** Proposed catalytic mechanism of acetone oxidation over the EMSI-modulated Pt_1_-CuO catalyst. Reprinted from Jiang et al. (2022). Copyright ^©^ 2022, with permission from Wiley-VCH GmbH. **(B)** Specific rate of CH_4_ conversion on Pt_1_/CoFe_2_O_4_ (Pt_1_/CF) and Pt_1_/CoFe_2_O_4_ after water treatment (Pt_1_/CF-W) at 300°C; The coordination structure of Pt_1_/CF **(C)** and Pt_1_/CF-W **(D)**. Reprinted from Yang Y. et al. (2022). Copyright ^©^ 2022, with permission from Springer Nature.

Strong metal-support interaction (MSI), as an important feature of SACs, can prevent the migration of isolated atoms and mediate electron transfer between metal atoms and supports (Weon et al., 2020; Yan et al., 2020). The adjustment of the electronic metal-support interaction (EMSI) is valid for modulating the catalytic performance of SACs (Hu et al., 2014; He et al., 2018). Jiang et al. (2022) manipulated EMSI between Pt and CuO to acquire dramatic increment of the performance on acetone oxidation. The strong EMSIs facilitates the charge redistribution through electron donation from Pt atoms to CuO, thus producing sufficient positively charged Pt species, which strengthen the adsorption/activation of acetone. Meanwhile, the activation of adjacent lattice oxygen, the crucial active oxygen species, is facilitated by the strong EMSI (Figure 3A).

However, the over-strong interaction between isolated atoms and supports could decrease the activity of SACs. The above-mentioned Pt_1_/CeO_2_ prepared by atom trapping showed excellent thermal stability, but it was inactive for CO oxidation at low temperatures due to the over-stabilized Pt^2+^ in a perfect square-planar Pt_1_O_4_ coordination (Jiang et al., 2021a). To break such strong interaction, Wang’s group developed a thermal-shock (TS) method to create an asymmetric Pt_1_O_4_ geometry, which was partially reduced into Pt_1_O_4-x_ unit during CO oxidation, forming active Pt_1_^δ+^ species to enhance low-temperature catalytic performance. Zhang’s group also found the MSI on Pt/CoFe_2_O_4_ was weakened by simple water soaking treatment and thus the C-H bond activation was enhanced in the catalytic combustion reaction of CH_4_ (Figure 3B) (Yang Y. et al., 2022). The critical function of water was disclosed to turn the coordination structure of Pt single atoms by forming Pt_1_-O(H^+^)-Fe unit. The coordinated H^+^ reduces the oxidation state of Pt and elongates Pt-O bond, so the MSI decreases (Figures 3C,D). This strategy is general for other metal-supported catalyst systems with MSI.

## 4 Characterizations of single-atom catalysts for air pollutant removal

With the fast-growing research on SACs, advanced characterization techniques have been developed to identify the existence, spatial distribution, coordination environment, electron structure and dynamic evolution of SACs. They can provide indispensable information to clarify structure-activity relationships and guide rational design of SACs. Diffuse reflectance infrared Fourier transform spectroscopy (DRIFTS), high-resolution microscopes, X-ray absorption spectroscopy (XAS) and theoretical calculations are “four treasures” to explore SACs.

**FIGURE 4:**
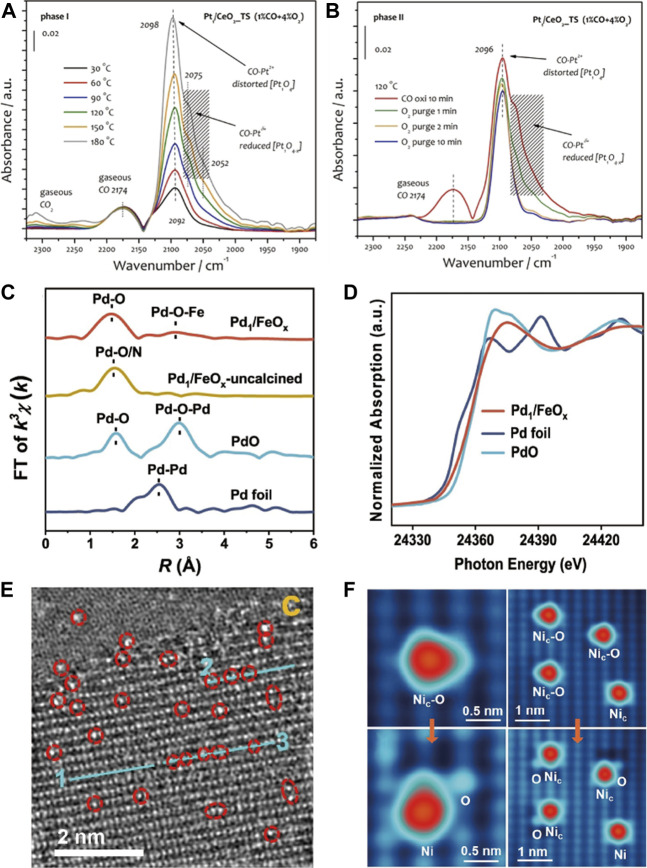
CO-DRIFTS of Pt_1_/CeO_2__TS catalysts **(A)** in CO oxidation, followed by O_2_/He purging **(B)**. Reprinted from Jiang et al. (2021b). Copyright ^©^ 2021, with permission from Wiley-VCH GmbH. The *k* ^3^-weighted Fourier-transform spectra from EXAFS **(C)** and normalized XANES spectra **(D)** at Pd K-edge. Reprinted from He et al. (2022). Copyright ^©^ 2022, with permission from Springer Nature. **(E)** HAADF-STEM image of Cu single-atoms anchored on TiO_2_. Reprinted from Zhang Y. M. et al. (2022). Copyright ^©^ 2022, with permission from Springer Nature. **(F)** STM image of “Ni_c_-O” species formed in O_2_ at room temperature and “Ni_c_-O” species can be split after pulsed-voltage manipulation. Reprinted from Zhou J. et al. (2022). Copyright ^©^ 2022, with permission from American Chemical Society.

DRIFTS using CO as a probe molecule is widely used to investigate the nature of supported platinum group metals (PGMs) (Pt, Pd and Au etc.) species (Jones et al., 2016; DeRita et al., 2017; DeRita et al., 2019; Jiang et al., 2020; Sarma et al., 2020), which can distinguish single atoms from nanoparticles by the vibration mode difference of adsorbed CO molecules. For example, the adsorption band of CO at 2,116 cm^−1^ is ascribed to the adsorption of CO over isolated Pt single atom of Pt_1_-Co_3_O_4_, whereas the band at 2089 cm^−1^ is caused by linear CO adsorption over metallic Pt nanoparticles of Pt/Co_3_O_4_ (Jiang et al., 2019). Meanwhile, the CO-DRIFTS can also monitor the evolution of SACs during the reaction. Wang’s group (Jiang et al., 2021b) observed a new shoulder bands at around 2075 and 2052 cm^−1^ during CO oxidation on Pt_1_/CeO_2__TS at temperatures ≥90°C, which was rapidly removed by O_2_ purging, indicating that Pt_1_O_4_ unit was partially reduced during CO oxidation (Figures 4A,B).

High-resolution microscopes can offer visual image to clearly verify the location of isolated atoms, particularly the high-angle annular dark-field scanning transmission electron microscope (HAADF-STEM), which can directly observe various metal atoms by Z (atomic-number) contrast between metal atoms and supports (Huang et al., 2021). Typically, for Cu-TiO_2_, the bright spots can be clearly viewed on the Ti atom rows, corresponding to Cu single atoms located at Ti vacancies (Figure 4E) (Zhang Y. M. et al., 2022). But it is out of operation when metal atoms are lighter than support atoms, such as Pd/CeO_2_ (Kim et al., 2021). Scanning tunneling microscope (STM) is also an atomic-level imaging technique, more than that, it can visualize the bonding of isolated atoms with reactant molecules. On Ni/CuO (Zhou J. et al., 2022), the adsorption behaviors of CO and O_2_ on Ni cations can be viewed under low-temperature STM. The isolated Ni cations (Ni_c_) are inert for CO adsorption and highly active for O_2_ dissociation at room temperature, resulting in“Ni_c_-O” species like isosceles triangles (Figure 4F).

X-ray absorption spectroscopy (XAS) based on synchrotron radiation can evaluate the coordination and electronic structures of SACs. Based on absorption energies, an XAS spectrum is divided into the X-ray absorption near edge structure (XANES) and extended X-ray absorption fine structure (EXAFS). XANES can give geometric structures and oxidation states of specific atoms, while the coordination environment, such as interatomic distance and coordination number, can be determined by EXAFS. For instance, on Pd1/FeO_x_, the normalized XANES spectra show that the absorption edge positions of Pd1/FeO_x_ were between those of Pd foil and PdO, suggesting positively charged Pd species (Figure 4D). In EXAFS, two prominent peaks at ∼1.5 and ∼2.9 Å originate from the Pd-O and Pd-O-Fe coordination, respectively, and Pd-Pd scattering is absent, excluding the possibility of the existence of Pd nanoparticles or clusters (Figure 4C) (He et al., 2022).

Theoretical calculations can assist to predict and confirm the coordination and electronic structures combining with XAS results (Qiao et al., 2011; Hoang et al., 2020). For example, Jin et al. studied the structure of Ni atoms on g-C_3_N_4_ substrate by XAS (Jin et al., 2020). EXAFS exhibited only a peak at about 2.0 Å, attributed to Ni-C/N bonding; and the fitting results revealed that the coordination number of Ni atoms was ∼5. Unfortunately, the similar scattering factor between C and N results made it difficult to distinguish the contributions from coordinated C and N atoms. Then, density functional theory (DFT) calculation was used to determine that Ni atom bonded with four N atoms and one C atom was the most stable structure.

## 5 Applications of single-atom catalysts for air pollutant removal

### 5.1 Carbon monoxide oxidation

CO is a toxic gas, mainly from automobile exhaust. CO oxidation at low-temperature has been the research focus. Additionally, CO oxidation, as an important model reaction, is often adopted to evaluate catalyst performance and reveal the underlying catalysis mechanism (Qiao et al., 2016; Dong et al., 2021).

CeO_2_ is regarded as an outstanding substrate because of its high redox ability and oxygen storage/release properties (Tan et al., 2020; Deng et al., 2022). CeO_2_ supported PGM single-atoms (such as Pt (Maurer et al., 2020), Au (Zhao et al., 2019), Pd (Kim et al., 2021), and Rh (Jeong et al., 2020a)) have been widely explored and used for CO oxidation due to their excellent activity, particularly Pt single-atoms loaded on CeO_2_ (Table 1). A lot of efforts have been made to improve the catalytic activity of Pt/CeO_2_.

**TABLE 1:** The catalytic performance comparison of SACs on CO oxidation.

Catalysts	Concentration (ppm)	Conversion (%)	Temperature (°C)	Ref
Pt/CeO_2_	19,000	90	64	Pereira-Hernandez et al. (2019)
Pt/CeO_2_	19,000	90	120	Wang et al. (2016)
Pt/CeO_2_	10,000	100	98	Wang et al. (2016)
Pt/CeO_2_	4,000	100	148	Nie et al. (2019)
Pt/CeO_2_	10,000	100	45	Jeong et al. (2020b)
Pd/Pr-CeO_2_	10,000	99	160	Deng et al. (2022)

**FIGURE 5:**
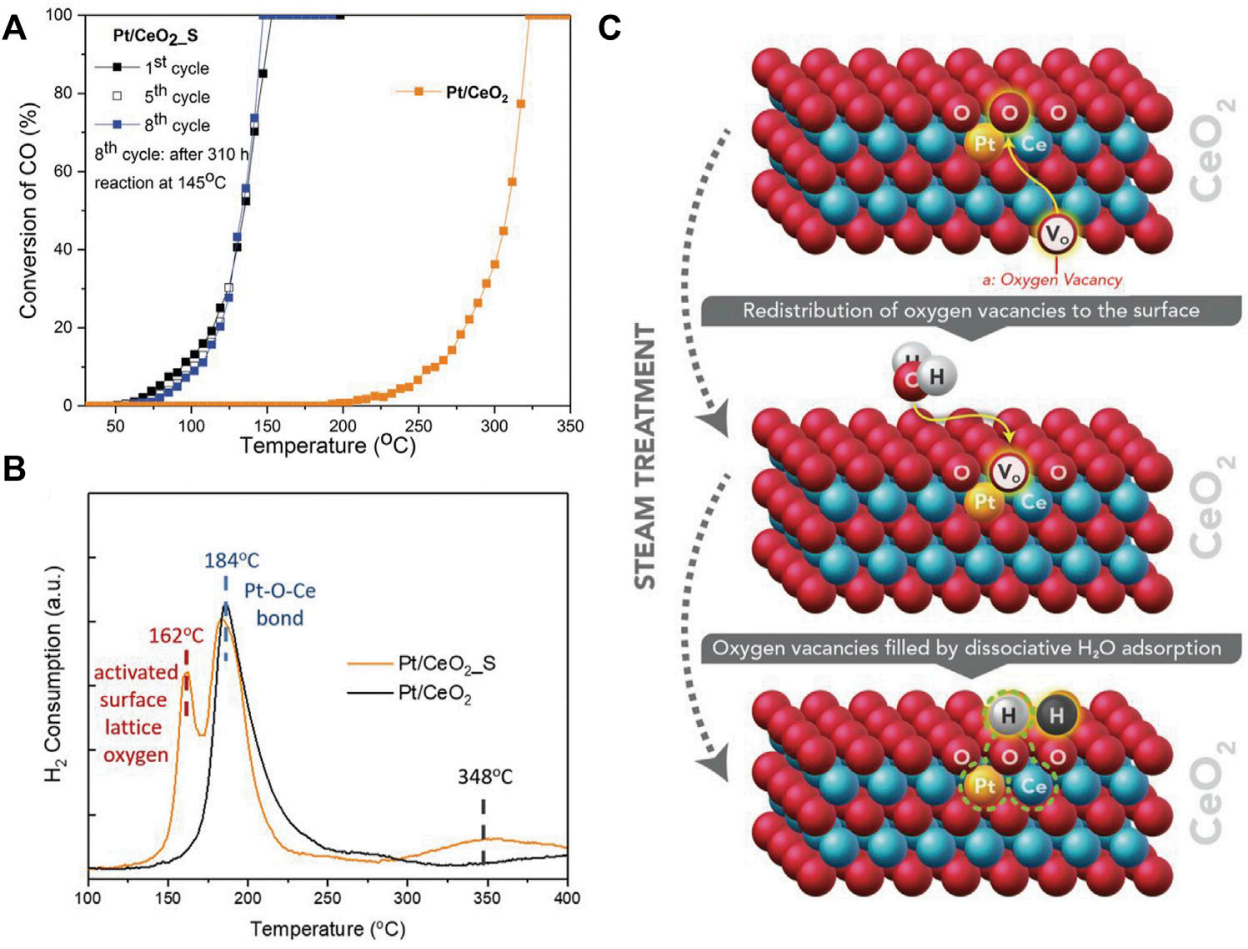
**(A)** CO oxidation performance and **(B)** H_2_-TPR profiles of Pt/CeO_2_ and Pt/CeO_2_ with steam treatment (Pt/CeO_2__S); **(C)** Illustration of active O_lattice_ [H] generated by steam treatment. Reprinted from Nie et al. (2019). Copyright ^©^ 2019, with permission from American Association for the Advancement of Science.

Varied preparation methods can lead to obvious difference in catalytic activity of Pt/CeO_2_ (Pereira-Hernandez et al., 2019). Pt/CeO_2_ prepared by atom trapping is more active than that obtained by wet chemical route because of the stronger interaction between Pt and CeO_2_, which can facilitate the reducibility of lattice oxygen. The presence of water can remarkably improve the CO conversion on Pt/CeO_2_ SACs by directly participating in CO oxidation (Wang et al., 2016). The adsorbed H_2_O can be easily dissociated to a hydroxyl (OH) and a lattice hydroxyl (O_lattice_H). Then CO reacts with the OH to yield a carboxyl intermediate, which dehydrogenates with the help of O_lattice_H to generate CO_2_ and water. This overall reaction route is more facile than the direct reaction of CO with lattice oxygen. Besides, the active O_lattice_ [H] derived from steam treatment at high temperature (750°C) can also activate Pt/CeO_2_, resulting in T_100_ dropping by 172°C compared with the untreated catalysts (Figure 5A) (Nie et al., 2019). H_2_-TPR analysis shows that new type of active surface lattice oxygen is generated during steam treatment (Figure 5B). It is revealed that H_2_O molecules can fill oxygen vacancies (V_O_) to produce two neighboring active O_lattice_ [H] in the vicinity of Pt single atoms under steam-treatment conditions (Figure 5C), providing dramatically enhanced catalytic activity. Different from the unstable surface OH from water dissociation, this active O_lattice_ [H] could be thermally stable up to 767°C.

The activity of Pt/CeO_2_ can also be promoted by H_2_ reduction treatment (Jeong et al., 2020b). The oxidation state of Pt single-atoms could be controlled by varying the reduction temperature. The metallic Pt SACs show higher activities than Pt NPs, whereas highly oxidized Pt SACs display poorer activities than Pt NPs. TPD measurements show the variation of oxidation state can modulate the adsorption strength of reactants and products, resulting in great disparity of catalytic performance.

The surface functionalization of CeO_2_ can boost thermal stability and catalytic activity of Pt/CeO_2_ in CO oxidation. The oxygen plasma pre-treatment on CeO_2_ surface has been demonstrated to serve as an effective strategy (Wan W. et al., 2022), which led to two crucial modifications of CeO_2_ surface: 1) surface restructuring, surface roughening that suppressing the diffusion and aggregation of Pt atoms; 2) a significant amount of surface peroxide (O_2_^2−^) powerfully anchoring Pt single atoms. These features are favor of a dense and uniform distribution of active Pt single atoms to boost CO oxidation.

### 5.2 Volatile organic compounds degradation

VOCs are a kind of organic compounds with boiling points in the scope of ∼50–260°C under ambient pressure (101.325 kPa) (Wu et al., 2021). In addition to the danger for human health, VOCs also participate in the formation of photochemical smog and secondary aerogel (Shayegan et al., 2018; Zheng et al., 2022). In view of the serious harm of VOCs, their efficient elimination by advanced oxidation techniques is of great significance. Recently, more and more SACs have been developed for VOCs degradation, e.g., formaldehyde, benzene and toluene (Table 2).

**TABLE 2:** The catalytic performance comparison of SACs on VOCs oxidation.

Catalysts		VOCs		Concentration (ppm)		Conversion (%)	Temperature (°C)	Light	Ref.
Ag_1_/HMO		HCHO		400		100	∼110	---	Hu et al. (2015)
Na_1_/HMO		HCHO		140		100	90	---	Chen et al. (2017b)
Au/α-MnO_2_		HCHO		500		100	75	---	Chen et al. (2018a)
Pt/Mn-TiO_2_		HCHO		100		100	15	---	Chen et al. (2019)
Pt1/CeO_2_		HCHO		400		100	15	---	Zhang et al. (2022a)
Ir1-N-C		HCHO		100		97	20	---	Peng et al. (2022)
Pt_1_/MnO_x_		C_7_H_8_		1000		90	219	---	Feng et al. (2022c)
Pt/MgO		C_7_H_8_		100		90	170	---	Zhao et al. (2020)
Pt/MnO_2_		C_7_H_8_		10		100	120	---	Zhang et al. (2019a)
Au-WO_3_/TiO_2_		C_7_H_8_		160		95.4	RT	300W Xe lamp	Qu et al. (2019)
Pt-MoS_2_/TiO_2_		C_7_H_8_		50		91.5	RT	LED (365 nm)	Wang et al. (2022)
Pt_1_/Fe_2_O_3_		C_7_H_8_		200		95	RT	720 mW/cm^2^	Wang et al. (2021)
Pt_1_/CuO-CeO_2_		C_7_H_8_		200		90	186	200 mW/cm^2^	Feng et al. (2022b)
Ag_1_/HMO		C_6_H_6_		200		100	220	---	Chen et al. (2017c)
Pd_1_Co_1_/Al_2_O_3_		C_6_H_6_		1000		90	250	---	Hou et al. (2021)
Pt_1_/meso-Fe_2_O_3_		C_6_H_6_		1000		90	198	---	Yang et al. (2019)
Ag_1_/Co_3_O_4_		C_6_H_6_		500		100	244	---	Fang et al. (2022)

#### 5.2.1 Formaldehyde degradation

Formaldehyde (HCHO) is the most prevalent indoor gaseous pollutant, which is released from construction and decoration materials (Yu and Crump, 1998; Salthammer et al., 2010). SACs for HCHO abatement consist of metal-oxide supported SACs (Hu et al., 2015; Chen et al., 2017b; Chen J. et al., 2018; Chen et al., 2019; Chen J. et al., 2020)and carbon supported SACs(Peng et al., 2022; Tang et al., 2022) according to the variety of supports.

In catalytic oxidation of HCHO, metal-oxide supported SACs usually obey the Mars-van-Krevelen (MVK) mechanism. Tang’s group (Hu et al., 2015) investigated the relationship between the electronic structure of single-atomic Ag active centers and the enhanced activity in HCHO oxidation. They found that the catalytic centers should include the single-atomic Ag sites and their vicinal lattice oxygen atoms, and their electronic states took a critical role. Single-atomic Ag sites with higher electron density facilitate the activation of O_2_, and the surface lattice oxygen with more negative charge has stronger nucleophilicity to oxidize HCHO. Subsequently, it is unveiled that the electronic environment of lattice oxygen is more significant than that of single-atomic metal sites in the catalytic centers for HCHO oxidation (Chen et al., 2017b). The surface lattice oxygen species of Na_1_/HMO is more negatively charged than that of Ag_1_/HMO, reaching higher efficiency in the oxidation abatement of HCHO.

Chen et al. (Chen J. et al., 2018) synthesized single-atomic Au doped α-MnO_2_ *via* the above-mentioned H_2_O_2_-assisted chemical etching method, removing HCHO of 500 ppm at 75°C and weight hourly space velocity (WHSV) of 60,000 ml/g∙h. Au single-atoms facilitate the formation of surface oxygen vacancies and the mobility of lattice oxygen. The same method was also conducted to prepare Pt/Mn-TiO_2_ (Mn doped TiO_2_) (Chen et al., 2019). Because of the strong interaction between Pt single atoms and Mn-TiO_2_ support, the redox properties of the catalyst, especially the reactivity of surface lattice oxygen, were improved, so the as-prepared catalyst exhibited enhanced performance on the low temperature oxidation of HCHO.

Zhang et al. (Zhang L. et al., 2022) adopted the strategy proposed by Nie et al. (Nie et al., 2019) to prepare Pt_1_/CeO_2_ with steam treatment, achieving 100% conversion of HCHO at 25°C. The O_lattice_H generated during the steam treatment was revealed as highly active sites for catalytic oxidation of DMO (dioxymethylene) and facilitates the further oxidation of formate species.

For HCHO oxidation over carbon supported SACs, Langmuir–Hinshelwood (L-H) mechanism is appropriate and molecular oxygen activation is pivotal. N-doped carbon stabilized iridium single atoms (Ir_1_-N-C) could deliver high HCHO removal efficiency (>95%) under high/low concentrations at 20°C (Peng et al., 2022). Ir single atoms, coordinating with four N atoms, serve as the active sites to adsorb and dissociate O_2_ by the strong electron coupling between Ir 5d orbital and O_2_ 2p orbital.

#### 5.2.2 Toluene oxidation

Toluene is a common VOC, emitted from decoration, furniture, and paint industries (Qu et al., 2019). Toluene with aromatic ring is harder to be degraded at low temperatures. Fortunately, the appearance of SACs has led to new advancement. In this section, the latest achievement on SACs for toluene oxidation is summarized, including thermo-catalysis, photocatalysis and photothermal catalysis.

For thermo-catalysis, toluene oxidation over SACs mainly follows the (MVK) (Zhang Y. et al., 2019; Wang et al., 2020; Feng et al., 2022c) or L-H (Zhang H. Y. et al., 2019; Zhao et al., 2020) mechanism. The crucial active oxygen species are surface lattice oxygen in MVK model. Feng et al. (Feng et al., 2022c) found the balance between lattice oxygen mobility and toluene adsorption improved the stability of Pt_1_/MnO_x_. The hydrogen reduction treatment can weak Mn-O bond and lower coordination number of Pt-O, causing superior mobility of lattice oxygen and appropriate toluene adsorption ability. Whereas, in L-H model, surface adsorbed oxygen is responsible for VOCs oxidation. Zhao et al. (Zhao et al., 2020) reported the introduction of H_2_O improved the catalytic activity of Pt/MgO catalyst in toluene oxidation and the T_90_ dropped by 50°C in humid environment than under dry conditions. On oxygen vacancies, dissociated molecular oxygen reacts with H_2_O to generate ∙OH, acting as dominant active oxygen species for toluene decomposition. Additionally, Zhang et al. (Zhang H. Y. et al., 2019) also disclosed that strong oxidative hydroxyl radicals (∙OH) on Pt/MnO_2_ surface contributed to excellent low-temperature catalytic activity at high space velocity. *In-situ* DRIFTS show that the vibration peaks of hydroxyl group negatively increase with reaction time, proving that the important role of surface hydroxyl.

On photocatalysis, noble metals, such as Pt, Au, and Ag, are ideal co-catalysts to boost catalytic activity. Wang et al. (2022) developed a simple two-step electrochemical approach to synthesize an atomically dispersed Au-loaded WO_3_/TiO_2_, achieving a 95.4% conversion and 85.5% mineralization rate for toluene removal. It is disclosed that Au single-atoms, anchored by oxygen vacancies on the WO_3_ surface, significantly enhanced the separation and transfer of photogenerated carriers and the adsorption of toluene. The increased MSI of the Au single atoms and WO_3_/TiO_2_ nanotubes ensures thermodynamic stability of Au single atoms. Qu et al. (2019) found that Pt sing-atoms can increase absorption of visible light and suppress the recombination of electron-hole pairs to improve photocatalytic performance on heterojunction system of Pt-MoS_2_/TiO_2_.

Photothermal catalysis is disclosed in two ways including light-driven thermocatalysis and photothermal synergistic catalysis. Light-driven thermocatalysis is actually thermocatalysis triggered by heat energy converted from light irradiation or solar energy. Wang et al. (Wang et al., 2021) synthesized Pt_1_/Fe_2_O_3_ catalyst with high light-driven thermo-catalysis performance, achieving toluene conversion of 95% and CO_2_ yield of 87% under the irradiation of 720 mW/cm^2^. It was ascribed to the high surface temperature stemming from excellent light-thermal conversion ability and the enhanced low-temperature reducibility by Pt single-atoms. Besides, the synergistic effect between photocatalysis and thermocatalysis over Pt_1_/CuO-CeO_2_ can achieve higher activity than individual thermocatalysis, with toluene conversion elevated by about 48% at 180°C (Feng et al., 2022b). Reactive oxygen species (∙OH and∙O_2_^−^) of photocatalysis further promote rapid transformation of the intermediates, accelerating release of the active sites. The Pt single-atoms could facilitate the utilization of photogenerated carriers and the adsorption/activation of oxygen molecules.

#### 5.2.3 Benzene oxidation

Nowadays, SACs are also employed on thermocatalytic oxidation of benzene (Chen et al., 2017c; Yang et al., 2019; Hao et al., 2021; Hou et al., 2021; Fang et al., 2022). Chen et al. (Chen et al., 2017c) dispersed Ag single-atoms on the surface of nanostructured hollandite manganese oxide (HMO), achieving 100% conversion at 220°C and high space velocity of 23,000 h^−1^. The reaction orders of benzene and O_2_ are ∼0.9 and ∼0.7, respectively, indicating the MVK mechanism is valid on Ag_1_/HMO. The activation ability toward both surface lattice oxygen and gaseous oxygen was improved after the loading of Ag single-atoms, accounting for its high catalytic activity. Dai’s group (Hou et al., 2021) prepared a Pd_1_Co_1_/Al_2_O_3_ single-atom catalyst for benzene oxidation and L–H model was suitable for this system. Pd site and Co site act as the benzene and oxygen adsorption sites, respectively, inhibiting their competitive adsorption. Furthermore, Pd_1_Co_1_/Al_2_O_3_ showed good sulfur resistance, which was attributed to the good regeneration ability of the active sites due to the rapid decomposition of sulfite or sulfate covered on Pd and Co sites.

### 5.3 Nitrogen oxides removal

NO_x_ is a sort of typical air pollutants mainly from fuel combustion and automobile exhausts (Gu M. L. et al., 2021) and severely impairs environment and human health, including acid rain, photochemical smog, global warming and respiratory diseases of humans (Boyjoo et al., 2017; Jiang et al., 2017; Nguyen et al., 2020; Pham VV et al., 2022). The emission control and purification treatment of NO_x_ are urgent problems to be solved.

**FIGURE 6:**
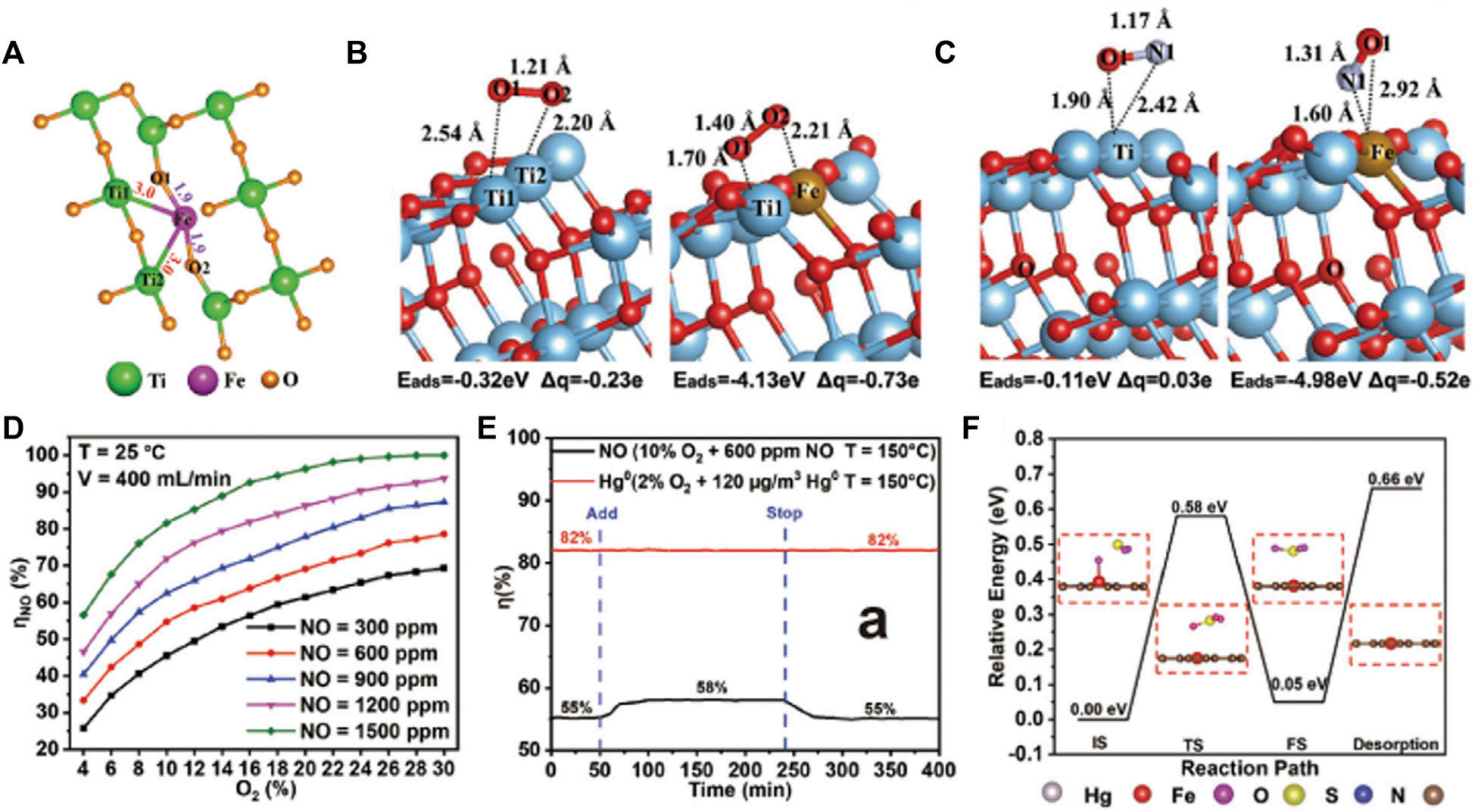
**(A)** Coordination structure of Fe single-atoms; adsorption models of **(B)** O_2_ and **(C)** NO on TiO_2_ before and after the anchor of single atomic Fe. Reprinted from Hu et al., 2020. Copyright 2020, with permission from Wiley‐VCH GmbH. **(D)** NO oxidation rates on Fe_1_-N_4_-C for varied concentrations of NO at 25°C; **(E)** NO oxidation rates on Fe_1_-N_4_-C with 500 ppm SO_2_ introduced; **(F)** Oxidation path between SO_2_ and the adsorbed O atom on Fe site of Fe_1_-N_4_-C. Reprinted from Yang et al., 2020b. Copyright 2022, with permission from Wiley‐VCH GmbH.

Photocatalysis is an effective technique to remove low-concentration (∼ppb level) NO_x_ at ambient temperature. NO_x_ adsorbed on catalyst surface can be converted into harmless nitrate *via* strong-oxidizing radical, such as superoxide radical and hydroxyl radical. Then, the generated nitrate is easily removed by simple washing to recycle photocatalysts (Boyjoo et al., 2017; Nguyen et al., 2020). Many SACs (e.g. Pt (Feng H. et al., 2022), Pd (Fujiwara and Pratsinis, 2017; Fujiwara and Pratsinis, 2018; Liu et al., 2021) and Fe (Hu et al., 2020) etc.) have been reported for photocatalytic NO_x_ removal (Table 3). Liu et al. (Liu et al., 2021) fabricated Pd single atoms confined by carbon vacancies of g-C_3_N_4_, displaying high activity and good stability in converting NO. The introduction of Pd single atoms restrains the recombination of photogenerated carriers and increases their average lifetime due to the low work function of Pd, allowing increased electrons to produce superoxide radical (∙O_2_^−^), which oxidizes NO to NO_3_^−^. Hu et al. (Hu et al., 2020) doped Fe single-atoms on Ti vacancies of TiO_2_ (Figure 6A), boosting the photo-reactivity towards NO oxidation and achieving NO removal rate as high as 47.91%. The electron transfer from Fe to Ti modulates the electronic structure of the bonded Ti atoms, resulting in the formation of dual active sites of Fe-Ti, which enhanced the activation of NO and O_2_ on Fe and Ti sites, respectively (Figures 6B,C). The synergistic effect of the dual active sites leads to a drastic promotion of photocatalytic oxidation of NO. In addition, the introduction of Fe single atoms sharply suppresses NO_2_ byproduct. But, during the process of NO oxidation, the generated nitrates would cover active sites to retard the catalytic performance. Therefore, high resistance to nitrate poisoning for SACs is demanded (Fujiwara and Pratsinis, 2018).

**TABLE 3:** The catalytic performance comparison of SACs on NO_x_ oxidation.

Catalysts	Concentration (ppm)	Conversion (%)	Temperature (°C)	Light	Ref
Pd/g-C_3_N_4_	2.2	56.3	RT	300 W Xe lamp	Liu et al. (2021)
Fe_1_/TiO_2_	50	47.9	RT	LED lamp (30W, λ > 400 nm)	Hu et al. (2020)
Pd/TiO_2_	1	50	RT	100 mW/cm^2^	Fujiwara and Pratsinis, (2017)
Fe_1_-N_4_-C	1,500	100	25	---	Yang W. J. et al. (2022)
Pd/θ-Al_2_O_3_	420	17	354	---	Narula et al. (2017)

The abatement of NO_x_ can also be accomplished by a two-step process, which involves an oxidation section to convert NO to NO_2_ and a reduction section to transform NO_2_ to N_2_. Jiang’s group (Yang W. J. et al., 2022) developed a single-atom Fe immobilized N-doped carbon catalyst (Fe_1_-N_4_-C), which showed ultrahigh catalytic activity for oxidizing NO to NO_2_ at low and room temperature (Figure 6D), owing to the low energy barriers of NO oxidation on Fe_1_-N_4_ sites. Moreover, Fe_1_-N_4_-C afforded robust stability against sulfur poisoning, benefiting from preferable adsorption of reactants rather than SO_2_ and SO_3_ on Fe_1_-N_4_ sites. Strikingly, the activity of NO oxidation was enhanced by 3% after introducing SO_2_ (Figure 6E), which was ascribed to that SO_2_ oxidation accelerated consumption of adsorbed O atoms on Fe_1_-N_4_ sites, thus leading to the regeneration of Fe_1_-N_4_ sites (Figure 6F).

## 6 Conclusions and perspectives

In this review, we introduce the synthesis and modulation strategies together with the characterization tools of SACs and their application in air pollutant removal, especially the abatement of CO, VOCs and NO_x_. It can be concluded that SACs have displayed immense superiority for effective treatment of various air pollutants because of its utmost atom utilization, tunable coordination and electron structure. Undoubtedly, satisfactory accomplishment of SACs has been made by persistent efforts; but it should be noted that SACs used in the field of air pollutant purification are still at the initial stage and face considerable challenges in fundamental research and practical engineering.

In terms of fundamental research of SACs, more attentions need to be paid to the following aspects. First, subtle difference of coordination and electron structure could cause distinct catalytic behavior despite identical single sites. So, the precise control to local environment of SACs has been a hard task. For non-noble metal single atoms, the increased metal loading conduces to improved catalytic activity. Although plentiful methods have been presented, the high content (>10wt%) of non-noble metal single atoms is still rarely reported. Besides, complex chemical reactions involving multiple reactants or reaction steps demand more than one active site to cooperate, resulting in that SACs are inactive for them. The synthesis of fully-exposed multi-atom catalysts has become a burning question. Furthermore, most synthesis approaches cannot receive high yields of SACs, which is averse to the demand of large-scale production in industry. So, facile and general synthesis approaches are highly desired. Apart from controllable synthesis, wide gaps are existing to in-depth understanding of structure-activity relationship of SACs during the air pollutant removal reactions. *Ex-situ* characterizations are still the main ways to explore the catalysis mechanism of SACs, resulting in great difficulties to detect the dynamic evolution of SACs during the catalytic reactions. With this in mind, *in situ*/operando techniques have risen to track dynamic changes of SACs in real reaction conditions. The enhancement of catalytic performance originates from the introduction of single atoms, so we focus on the change of single atoms during the *in situ*/operando characterization. However, for traditional nanoparticles or clusters, many factors need to be considered, such as the role of multiple active atoms and interface effect etc. *In situ* TEM/STM can directly observe surface morphology and local structure changes around isolated atoms. *In situ* XAS offers real-time electron and coordination structures of SACs and AP-XPS provides valence state information of SACs during catalytic reactions or under quasi-conditions of reactions. But *in situ* microscope only gets local information of fixed area; the beam time of *in situ* XAS is restricted and AP-XPS suffers from low atom-resolution. Hence, limitations of each techniques make them still far from enough to decode structure-performance relationship of SACs and tight integration of various *in situ* techniques is essential to unlock the black boxes of reaction mechanisms. On the other hand, due to the advantages of the well-defined single atomic site, calculation model of SACs is closer to the actual configuration; therefore, SACs are ideal model systems to operate theoretical study for gaining atomic-level insight into catalytic reaction mechanisms. Nowadays, theoretical calculations are easily conducted for small molecules, e.g., CO and HCHO. However, the adsorption and reaction behaviors of large molecules (e.g., C_6_H_6_ and C_7_H_8_) on SACs seem to be complicated. Because of the more possible configurations of these larger molecules on catalyst surface, the complication involves the establishment and search of adsorption active sites, transition states and intermediates, together with the calculation of reaction barriers. In-depth research closely combining *in-situ* explorations and DFT calculations will bring new understanding on catalysis mechanism. On the modulations of the coordination environment and electron structures, little efforts have been devoted. The nature of supports has significant effects on the chemical environment of SACs; and single atoms usually work together with the surrounding atoms of support to achieve high catalytic performance in some reactions. Besides, rational regulations on the MSI effects of SACs have been confirmed as very crucial issues to optimize their catalytic performance. Hence, the function of supports deserves more concerns in the future research; additionally, the generic descriptor of MSI effects demands to be established to screen optimal combination of metal atoms and supports.

For practical applications, SACs also confront a serious of challenges. Apart from single pollutant in laboratory, real gas mixtures to be treated probably contain some halogenated or sulfur-containing pollutants, which react with catalysts to cause their deactivation. Thus, high demands are imposed for improving the resistance ability of SACs to poison components. Another challenge is to ensure stability of SACs during long-term operation. High-temperature and reaction media may induce migration of isolated atoms, then inhibit the catalytic activity of SACs. So, how to maintain atomic dispersion of metals on SACs under working conditions is worth careful considerations. Finally, in available literatures, in most cases SACs work in powder form; but industrial catalysis requires monolithic catalysts and set reaction devices. Therefore, it can be predicted that the integration of SACs into set reactors will be a top priority for their large-scale applications.
